# OBMeta: a comprehensive web server to analyze and validate gut microbial features and biomarkers for obesity-associated metabolic diseases

**DOI:** 10.1093/bioinformatics/btad715

**Published:** 2023-12-11

**Authors:** Cuifang Xu, Jiating Huang, Yongqiang Gao, Weixing Zhao, Yiqi Shen, Feihong Luo, Gang Yu, Feng Zhu, Yan Ni

**Affiliations:** Children’s Hospital, Zhejiang University School of Medicine, National Clinical Research Center for Child Health, Hangzhou, Zhengjiang 310052, China; Children’s Hospital, Zhejiang University School of Medicine, National Clinical Research Center for Child Health, Hangzhou, Zhengjiang 310052, China; Department of Epidemiology and Health Statistics, Zhejiang University School of Public Health, Hangzhou, Zhengjiang 310058, China; Children’s Hospital, Zhejiang University School of Medicine, National Clinical Research Center for Child Health, Hangzhou, Zhengjiang 310052, China; Children’s Hospital, Zhejiang University School of Medicine, National Clinical Research Center for Child Health, Hangzhou, Zhengjiang 310052, China; Department of Epidemiology and Health Statistics, Zhejiang University School of Public Health, Hangzhou, Zhengjiang 310058, China; College of Agriculture and Biotechnology, Zhejiang University, Hangzhou, Zhengjiang 310058, China; Department of Pediatric Endocrinology and Inherited Metabolic Diseases, Children's Hospital of Fudan University, Shanghai 201102, China; Department of Data and Information, Children’s Hospital, Zhejiang University School of Medicine, Hangzhou, Zhengjiang 310052, China; College of Pharmaceutical Sciences, Zhejiang University, Hangzhou, Zhengjiang 310058, China; Children’s Hospital, Zhejiang University School of Medicine, National Clinical Research Center for Child Health, Hangzhou, Zhengjiang 310052, China; Department of Epidemiology and Health Statistics, Zhejiang University School of Public Health, Hangzhou, Zhengjiang 310058, China

## Abstract

**Motivation:**

Gut dysbiosis is closely associated with obesity and related metabolic diseases including type 2 diabetes (T2D) and nonalcoholic fatty liver disease (NAFLD). The gut microbial features and biomarkers have been increasingly investigated in many studies, which require further validation due to the limited sample size and various confounding factors that may affect microbial compositions in a single study. So far, it lacks a comprehensive bioinformatics pipeline providing automated statistical analysis and integrating multiple independent studies for cross-validation simultaneously.

**Results:**

OBMeta aims to streamline the standard metagenomics data analysis from diversity analysis, comparative analysis, and functional analysis to co-abundance network analysis. In addition, a curated database has been established with a total of 90 public research projects, covering three different phenotypes (Obesity, T2D, and NAFLD) and more than five different intervention strategies (exercise, diet, probiotics, medication, and surgery). With OBMeta, users can not only analyze their research projects but also search and match public datasets for cross-validation. Moreover, OBMeta provides cross-phenotype and cross-intervention-based advanced validation that maximally supports preliminary findings from an individual study. To summarize, OBMeta is a comprehensive web server to analyze and validate gut microbial features and biomarkers for obesity-associated metabolic diseases.

**Availability and implementation:**

OBMeta is freely available at: http://obmeta.met-bioinformatics.cn/.

## 1 Introduction

Obesity is now a global health issue with rapidly increased prevalence that reached 39% worldwide and affected almost 2.5 billion people according to the World Health Organization (WHO) report in 2016 (Chooi *et al.* 2019). Previous studies have showed that obesity increases the risk of other metabolic diseases, including type 2 diabetes (T2D) (Franks and McCarthy 2016), nonalcoholic fatty liver disease (NAFLD) (Riazi *et al.* 2022), and metabolic syndrome (encompassing hypertension, dyslipidemia, and insulin resistance) (DeMarco *et al.* 2014, Khan *et al.* 2018). The epidemic of these global obesity-associated diseases causes extensive social and economic implications, so effective intervention is necessary.

Gut microbiota is a complex community of microorganisms inhabiting the host gastrointestinal tract that has crucial roles in host health maintenance (Jandhyala *et al.* 2015). Increasing evidence has demonstrated that dysbiosis of gut microbiota propels the emergence of obesity and correlates with other metabolic diseases. For example, lipopolysaccharides (LPS) derived from pathogenic bacterial membranes can trigger chronic inflammation in T2D and obesity (Qin *et al.* 2012, Boulangé *et al.* 2016). The proliferation of the short-chain fatty acids-producing bacteria benefits weight loss and enhances insulin sensitivity (Tolhurst *et al.* 2012, Marques *et al.* 2018). In addition, different kinds of weight loss strategies, such as exercise, medication, probiotics intervention (Wang *et al.* 2015), vegetarian diet (Tomova *et al.* 2019), and even stomach surgery (Ulker and Yildiran 2019), can reshape gut microbiota. Therefore, identifying reliable and consistent microbial biomarkers is essential helping us to explore prospective therapeutic targets for obesity-associated metabolic diseases.

With the widespread application of next-generation sequencing technologies in the biomedical field, accumulating number of obesity-associated datasets of gut microbiome has been published in public databases. Metagenomic analyses are challenging due to the large volume and complex nature of raw sequencing data. For nonbioinformatics experts, it is demanding to apply computational programming and algorithms for high-throughput data processing and analysis. Recently, to make more available of comparative metagenomics analysis for clinicians and bench researchers, several powerful web servers have been developed, such as MG-RAST (Wilke *et al.* 2016), METAGEN-assit (Arndt *et al.* 2012), MicrobiomeAnalyst (Lu *et al.* 2023), and Busybee (Schmartz *et al.* 2022). They offer efficient microbial data processing, analysis, and visualization.

After initial biomarker discovery, further validation is important due to the limited sample size in an individual study and various confounding factors that may affect preliminary findings. Since it is time/cost-consuming to conduct independent studies, applying bioinformatics techniques to analyze publicly available metagenomic datasets is preferred in order to validate reliable biomarkers more efficiently. So far, it lacks a comprehensive bioinformatics pipeline providing automated statistical analysis and cross-project validation with comparable studies simultaneously. To identify and validate the microbial biomarkers of obesity associated metabolic diseases, we have developed a comprehensive and automated data analysis platform named OBMeta, which integrates microbial diversity analysis, comparative analysis, functional analysis, and co-abundance network construction. In addition, OBMeta provides cross-project, cross-phenotype, and cross-intervention validation based on the curated database. It helps users to identify the consistent microbial features and reliable biomarkers of obesity-related metabolic diseases efficiently.

## 2 Materials and methods

### 2.1 Data collection and curation

Raw sequencing reads (including 16S amplicon raw reads and metagenomic raw reads) were downloaded from NCBI SRA (Sequence Read Archive) (Kodama *et al.* 2012) and EBI ENA (European Nucleotide Archive) (Harrison *et al.* 2019) databases using transfer tool Aspera. Sequencing related metadata and host related metadata were obtained from these databases or published paper, respectively. We manually included the datasets based on the following criteria: datasets collected from biological samples (e.g. feces, intestine, oral, skin, etc.) of humans and animals; studies with at least one single-factor comparison on obesity-associated metabolic diseases (i.e. obesity, T2D, or NAFLD); raw data can be linked to at least one published paper.

### 2.2 Processing of raw data

Supplementary Figure S1 illustrates the detailed pipeline for constructing the curated database of OBMeta. We evaluated the overall quality of the downloaded datasets using FastQC and then processed the 16S amplicon and metagenomic raw data, respectively. Supplementary Table S1 summarized the software or algorithms during the data processing.

For the 16S sequences, QIIME 2 (Hall and Beiko 2018) and DADA 2 (Prodan *et al.* 2020) were applied for processing sequences and denoising respectively. The output from DADA 2 was clustered into 99% identity using an operational taxonomic unit (OTU) picking protocol against the Sliva 132 database. Functional annotations were conducted by PICRUSt2 (Douglas *et al.* 2020) with default parameters.

For metagenomic sequences, the sequencing vectors and low-quality bases was removed by Trimmomatic (Bolger *et al.* 2014). The clean reads were mapped against the host reference genome using Bowtie 2 (Langmead and Salzberg 2012) to filter out the host contamination. The high-quality reads of single sample were first assembled by MEGAHIT (Li *et al.* 2016). Next, QUAST (Gurevich *et al.* 2013) was applied to evaluate the quality of the assembling. Then, the assembled reads were predicted open reading frames (ORFs) and filtered (<100 nt) by MetaGeneMark (Hyatt *et al.* 2012). The qualified reads were translated into amino acid sequences and clustering into nonredundant contigs with 95% identity and 90% coverage using CD-HIT. Finally, Diamond was used to annotate different taxonomies and functional pathways against NR and KEGG databases with an e-value cutoff of 1E-5, respectively. Profiles of different taxonomic relative abundance and functional abundance were determined by Salmon (Patro *et al.* 2017).

### 2.3 Consistency evaluation of microbial changes among different projects

To evaluate the consistency of a significantly varied taxon among different comparisons, we defined a score by the formula:

$$\mathrm{Consistency}\;\mathrm{score}\;\left(\mathrm{CS}\right)=\frac{n1-n2}{\mathrm{Number}\;\mathrm{of}\;\mathrm{comparisons}\;\#},$$

where n1 is the number of intergroup comparisons with a statistically significant increase in a case group; n2 is the number of intergroup comparisons with a statistically significant decrease in a case group; # indicates statistically significant comparison.

The value of CS score (ranging from −1 to 1) indicates the consistency degree of a taxon across different projects. The CS value close to 1 or −1 indicates the higher degree of consistent up or down-regulation. To be noted, a single project may include multiple comparisons, e.g. different time points or dosages, thus the number of comparisons is considered for evaluation indeed. In addition, considering the degree of changes between two groups to ensure that a potential biomarker with a larger fold change will receive a higher score, a weighted consistency score (WCS) is calculated as follows:

$$\mathrm{Weighted}\;\mathrm{consistency}\;\mathrm{score}\;\left(\mathrm{WCS}\right)=\frac{\sum_{n=1}^{i}\left(a_{i}{\mathrm{Log}}_{2}{\mathrm{FC}}_{i}\right)}{\sum_{n=1}^{i}\left(\left|{{\mathrm{Log}}_{2}FC}_{i}\right|\right)},$$

where *i* is the comparison with a statistically significant change and *a* is the coefficient 1 or −1 for up- or down-regulation of taxon abundance.

### 2.4 Software development and implementation

OBMeta is implemented as a web server using JavaScript, HTML, and cascading style sheets for fronted development. The core JavaScript library Vue.js (https://vuejs.org/) and Spring Boot (https://spring.io/projects/spring-boot) were used as the main frontend and backend frameworks, respectively. The interactive interface was implemented using WebSocket. R in-house scripts were used for backend data processing, analyses, and visualizations. Open-source data management system MySQL (https://www.mysql.com/) is used for data persistent storage. OBMeta was deployed on Dell PowerEdge Server in the Children’s Hospital, Zhejiang University School of Medicine with 32 virtual CPUs (2.10 Hz), 256 GB memory, and 30T solid-state drives.

## 3 Results

**Figure 1.**
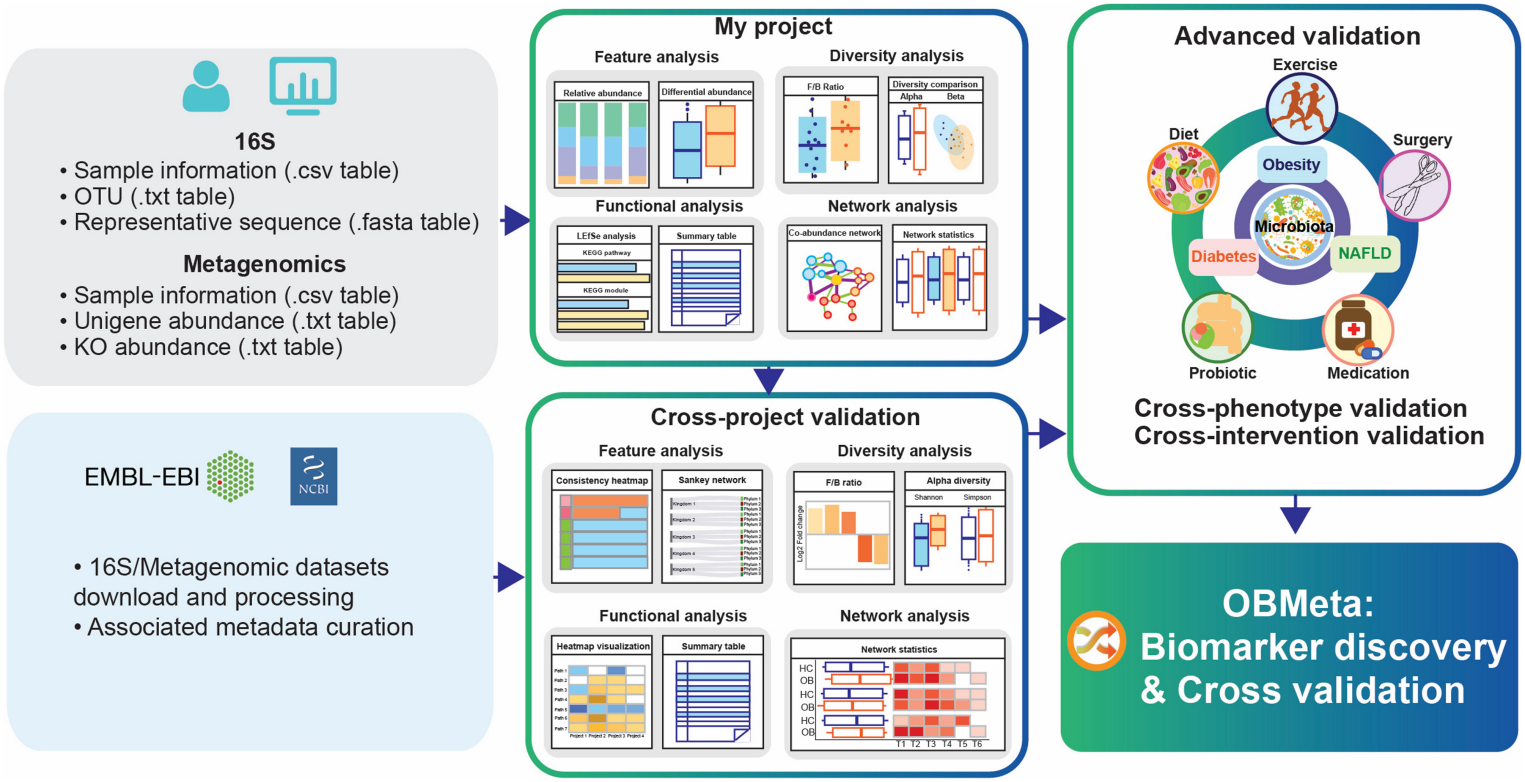
The workflow and functional features of OBMeta.

OBMeta is designed with three modules: (i) my project analysis, (ii) cross-project validation, and (iii) advanced validation. The overall workflow and analysis strategy are summarized in Fig. 1 and Supplementary Fig. S2. The first module provides the comparative analysis including feature analysis, diversity analysis, functional analysis, and network analysis. The second module is to perform cross-project validation of gut microbial features across selected projects. The last module provides advanced validation for cross-phenotype or cross-intervention comparisons. All the results from main modules are available to download for further analysis and interpretation.

### 3.1 My project analysis

#### 3.1.1 Data uploading and filtering

16S rRNA sequencing and shotgun metagenomics are the most common sequencing strategies to characterize microbial compositions. OBMeta is designed to accept 16S rRNA marker gene data and metagenomics data. To perform compositional comparative analysis, two files containing sample information and abundance records are required. In addition, OBMeta provides functional annotation and interpretation if representative sequence or KO (KEGG Orthology) abundance datasets are available. OBMeta is compatible with taxonomy outputs from QIIME2 (Bolyen *et al.* 2019), Kraken2 (Wood *et al.* 2019), and MetaPhlAn4 (Blanco-Míguez *et al.* 2023), as well as functional annotation datasets from PICRUSt2 (Douglas *et al.* 2020), Tax4Fun (Wemheuer *et al.* 2020), and HUMAnN3 (Beghini *et al.* 2021) (Supplementary Table S3).

After uploading the data files, OBMeta will process the missing data automatically following the predefined steps: (i) to exclude features with missing values within more than 80% of all the samples; (ii) to fill the zero with the 1/10 of minimum abundance; and (iii) to normalize data by total-sum scaling (TSS) for 16S data. For metagenomics data input, OBMeta will produce gene abundance with transcripts per million (TPM) normalization using Salmon. This automatic filtering procedure can improve the statistical power and provide more robust results for downstream analysis. In practice, users have the flexibility to decide the extent to which missing values should be filtered. Subsequently, they can apply further normalization using the median of ratios method in DESeq2, rarefying to the minimum library size, or utilizing the TSS.

#### 3.1.2 Standardized analysis for local datasets

**Figure 2.**
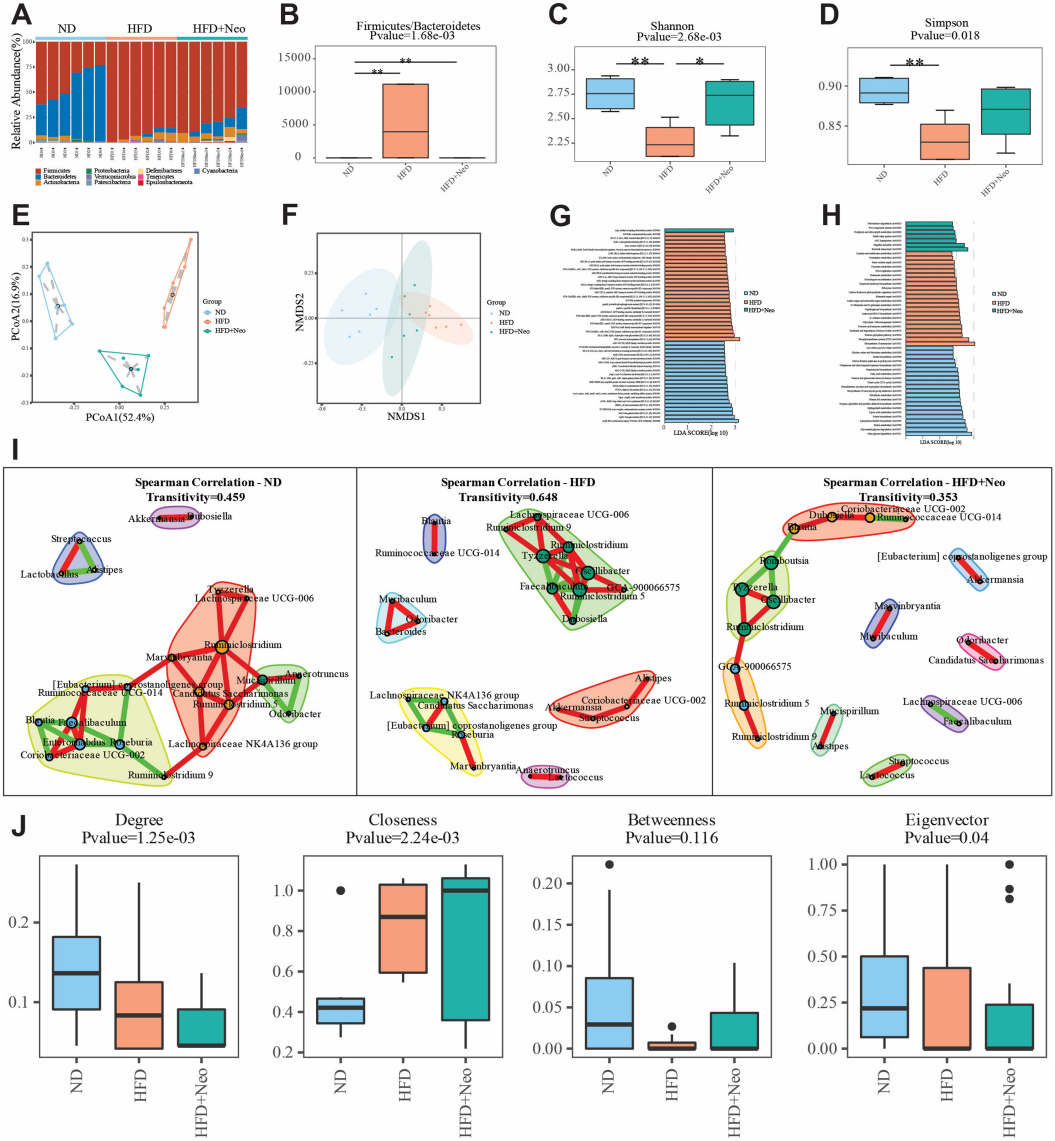
Outputs of the example datasets from “My project” module of OBMeta. (A) A stacked bar plot of the relative abundance of gut microbiome at the genus level. (B) The Firmicutes/Bacteroidetes ratio of each group. (C) A bar plot of the Shannon index based on genus profile. (D) A bar plot of the Simpson index based on genus profile. (E) The ordination plot based on the Bray–Curtis distance at genus level using principal coordinate analysis (PCoA). (F) The ordination plot based on the Bray–Curtis distance at genus level using nonmetric multidimensional scaling (NMDS). (G) A bar plot of KO modules with significant LDA score (>2) in LEfSe analysis (top 50 most significant modules). (H) A bar plot of functional pathways with significant LDA score [Log10(LDA) > 2] in LEfSe analysis (top 50 most significant pathways). (I) The co-abundance network from Spearman’s correlation analysis based on the top 30 most abundant genera for each group. (J) The box plots of the network properties including degree, closeness, betweenness, and eigenvector.

This module allows users to analyze their own datasets in four steps including feature analysis, diversity analysis, functional analysis, and network analysis. In the feature analysis section, the OTU tables from marker gene or unigene can be assigned to different taxonomic level before conducting comparisons. The overview of microbial features is visualized as stacked bar plots (Fig. 2A). OBMeta provides nonparametric univariate analysis by default for differential feature analysis (Kruskal-Wallis test, Supplementary Fig. S3), with other alternative options including LEfSe (Paulson *et al.* 2013), ANCOMBC (Lin and Peddada 2020), and DESeq2 (Love *et al.* 2014).

The Firmicutes/Bacteroidetes (F/B) ratio is widely considered as an important marker of intestinal homeostasis (Stojanov *et al.* 2020). Increased F/B ratio is regarded as a potential signature in obesity (Magne *et al.* 2020). So OBMeta offers the F/B ratio comparison among different groups (Fig. 2B). In addition, the alpha-diversity is calculated at different taxonomic levels using Shannon index and Simpson index, which emphasizes the richness component and evenness component of diversity (Fig. 2C and D). The beta-diversity analysis provides two common distance measures (Bray–Curtis distance and Jaccard distance), which are represented by 2D ordination plots based on principal coordinate analysis (PCoA) or nonmetric multidimensional scaling (NMDS) (Fig. 2E and F). The corresponding statistical significance is assessed using ANOSIM test (Supplementary Fig. S4).

In the functional analysis, OBMeta predicts the functional profiles (KO modules and pathways) for 16S rRNA data or metagenomics data. Nonparametric univariate analysis and linear discriminant analysis (LDA) effect size (LEfSe) (Segata *et al.* 2011) method are applied to identify the significant features respectively, and the example outputs from LEfSe were presented in Fig. 2G and H.

In the microbial community, ecological interactions between constituent taxa are vital in determining the overall structure and function of the community in host health and disease (Loftus *et al.* 2021). Also, the microbial composition varies significantly between individuals raising the question whether there is a core microbiome that produces main effect with respect to the healthy and diseased states. To disclose the core compositions in the gut microbes, the network analysis module is applied to construct the bacterial association network and calculate the network topological properties for each group based on their different taxonomic levels. The network nodes are colored according to the network modularity and the node sizes are proportionated with the degree (Fig. 2I). The network properties (degree, closeness, betweenness, and eigenvectors) are compared using the Wilcoxon rank-sum test (Fig. 2J).

### 3.2 Cross-project validation

#### 3.2.1 Cross-project comparison and validation

The cross-project validation module allows users to identify the consistent microbial features from different comparisons in the curated database. The curated database now includes 234 comparisons classified by phenotypes (case versus control) and intervention strategies (case with intervention versus case without intervention/before intervention versus after intervention) from a total of 90 projects in obesity, T2D, and NAFLD. Users can search similar projects according to the disease type, intervention strategy, and other filtering conditions including age, gender, species type, sample type, and sequencing platform.

**Figure 3.**
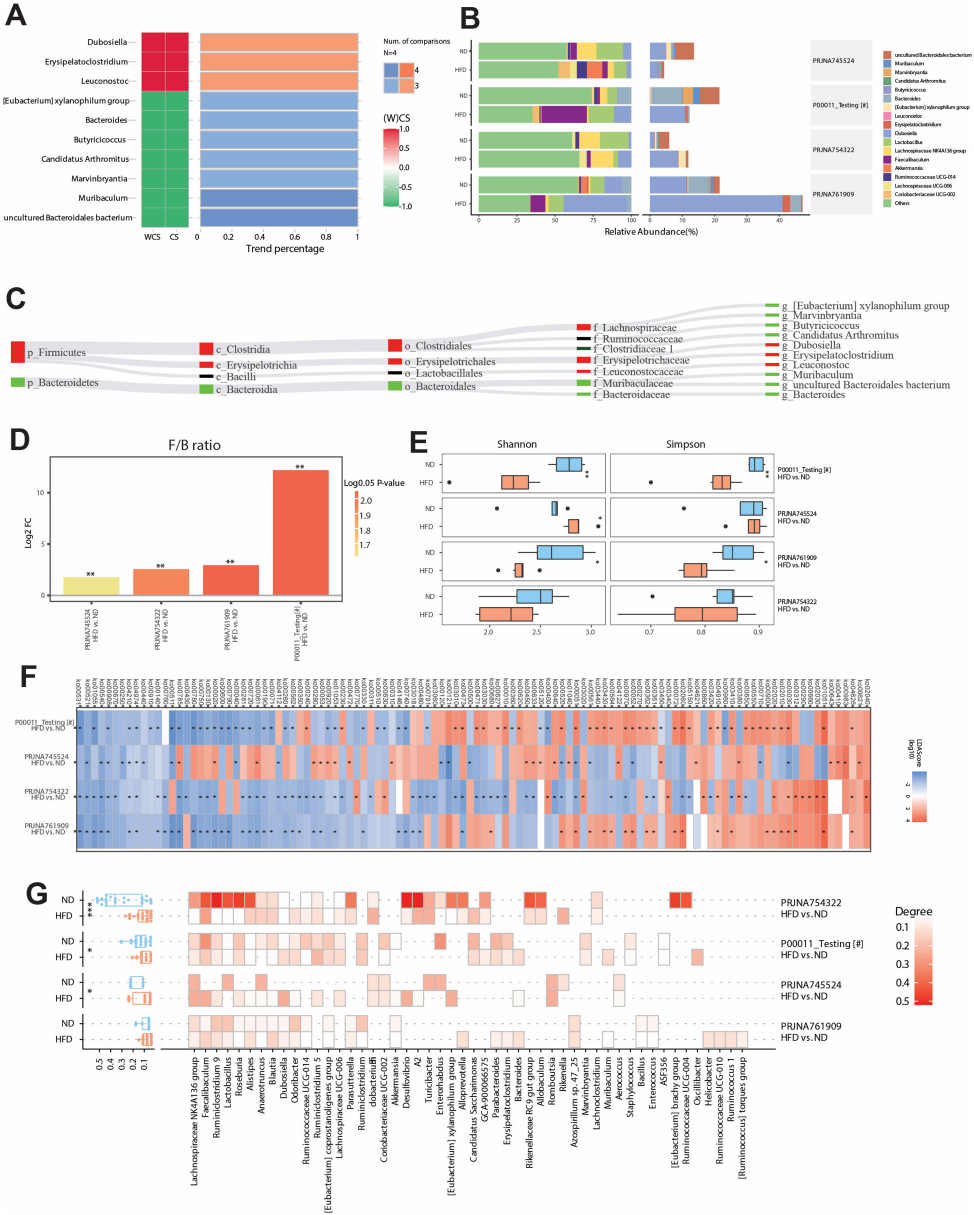
Outputs of the cross-project validation using the example comparison in “Phenotype” classification. (A) Consistency heatmap of the significantly changed bacteria at the genus level among the selected projects. The heatmap showed all phyla that significantly varied in at least 3 projects with the consistency score more than 0.6. (B) The stacked bar plot of the relative abundance of top 10 abundant genera and others (the left panel) and the consistently varied genera (the right panel). Local comparison was labeled with #. (C) Sankey network of the consistently varied genera that met the defaulted conditions. (D) The bar plot of the fold changes of the Firmicutes/Bacteroidetes ratio among different projects. It is colored based on the log2 transformation of *P*-value in the comparison. (E) The box plots of alpha diversity (Shannon and Simpson indices based on the genus profile) in each group. (F) The heatmap of the significant pathways [log10(LDA) > 2, LEfSe analysis] in any one of the selected projects. *, *P* < 0.05 (G) The heatmap of the network property (degree) in each group based on the top 50 most abundant genera.

Similarly, there are four steps of automated statistical analysis. In the feature analysis, OBMeta evaluates the consistent variation of taxa using CS and WCS (Fig. 3A), compositional proportion calculation using stacked bar plot (Fig. 3B), and Sankey network (Fig. 3C) at different taxonomic levels. In the diversity analysis, OBMeta presents the cross-project comparison of the F/B ratio (Fig. 3D) and alpha diversity (Fig. 3E). In the functional analysis, OBMeta summarizes the pathways with significant LDA scores [log10(LDA) > 2, *P *<* *0.05] in each project (Fig. 3F). In the network analysis, OBMeta calculates the network properties based on the top 30 most abundant taxa (Fig. 3G). These multidimensional comparisons help to efficiently identify the consistent microbial signatures for obesity-associated metabolic diseases.

### 3.3 Advanced validation

#### 3.3.1 Advanced validation via multi-disease and multi-intervention comparisons

**Figure 4.**
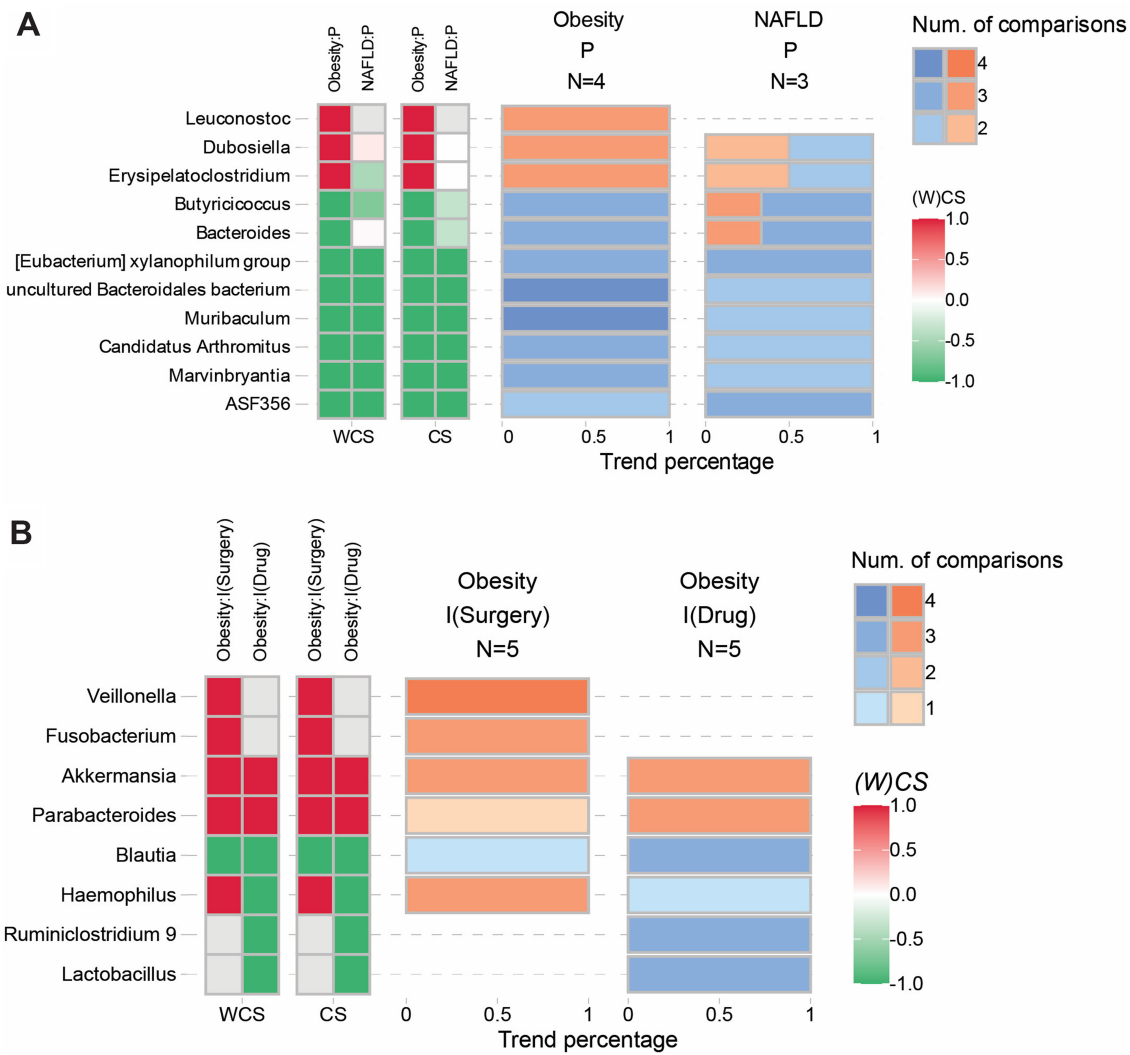
Example outputs of the advanced validation. (A) Consistency heatmap of the significant phyla between obesity and NAFLD. (B) Consistency heatmap of the significant phyla between the drug (natural extracts) and diet intervention with positive effects on obesity.

Obesity is closely associated with other metabolic diseases, and various medical, nutritional, or physical interventions have been conducted targeting the gut microbiota. Therefore, OBMeta offers multi-disease and multi-intervention evaluations of varied taxa in the module of advanced validation. The own project and selected projects for cross validation will be integrated as a whole by default to compare with other phenotypes (Fig. 4A) or interventions (Fig. 4B).

OBMeta is designed flexibly that allows users to stop or restart their analysis in each module. If users do not have their own datasets for analysis and validation, publicly available datasets within OBMeta are also valuable for microbial signature analysis of a certain phenotype or intervention.

### 3.4 Case study I

To illustrate the utility of OBMeta, we chose a publicly available project of an animal experiment (PRJNA601832). This is a study on the beneficial effect of Neohesperidin (Neo, a natural polyphenol abundant in citrus fruits) in obesity using 8-week-old male wildtype (C57BL/6J) mice. Fecal samples were collected for 16s amplicon sequencing analysis after 12-weeks of normal diet (ND), high-fat diet (HFD), and high-fat diet plus Neo (HFD + Neo). Two pairwise comparisons were conducted (HFD versus ND and HFD + Neo versus HFD) to validate the gut dysbiosis of obese mice and effects of Neo intervention, respectively.

In the feature analysis section of “My project” module, we found that the phylum *Firmicute* significantly increased and *Bacteroidetes* decreased in the HFD group while Neo intervention can restore these microbial disturbances to some extent (Fig. 2A). The comparative analysis further showed that *Leuconostoc*, *Faecalibaculum*, uncultured *bacterium*, *Dubosiella*, *Globicatella*, and *Candidatus arthromitus* were the top differential genera among three groups (Supplementary Fig. S3). In the diversity analysis section, the *Firmicutes*/*Bacteroidetes* ratio was significantly increased in the HFD group (Fig. 2B). Alpha diversity indices (Shannon and Simpson index) were obviously decreased in the HFD group compared to the ND and HFD + Neo groups (Fig. 2C and D). The community structure also significantly varied according to the ordination plots (Fig. 2E and F) and ANOSIM test (Supplementary Fig. S4). In the functional analysis section, the significant KO modules and functional pathways were provided in each group (Fig. 2G and H). In the network analysis section, the network plot indicates the core bacterial connections for each group (Fig. 2I). For example, the beneficial genus *Akkermansia* was positively correlated with *Dubosiella* in the ND mice while was positively correlated with *Streptococcus* in the HFD mice. The comparison of network properties also showed the degree and eigenvector were significantly decreased in the HFD network which indicated less connections at the genus level, while their closeness was significantly increased in HFD network (Fig. 2J).

Furthermore, to validate the microbial features induced by high fat diet (HFD versus ND), we selected three more similar projects (Supplementary Table S2) to evaluate their consistent variation among different projects. As shown in Fig. 3A, three genera were consistently and significantly increased (e.g. *Dubosiella* and *Erysipelatoclostridium*) as well as seven genera were decreased (e.g. *Eubacterium* and *Bacteroides*) in the high-fat-diet mice of more than three projects. The stacked bar plot indicated the relative expressions of microbes specifically in each comparison (Fig. 3B). We can find *Dubosiella* and *Lactobacillus* were enriched in all comparisons. The Sankey network demonstrated the consistently varied genera and their taxonomic classification (Fig. 3C). In the diversity analysis, the *Firmicutes*/*Bacteroidetes* ratio showed consistently increased in selected comparisons (Fig. 3D) and significantly decreased alpha diversity indicated by Shannon and Simpson indices in both own project and PRJNA761909 (Fig. 3E). The function analysis further showed that the pathways Ko00908 (zeatin biosynthesis), Ko04974 (protein digestion and absorption), and Ko04210 (apoptosis) were consistently decreased in four projects (Fig. 3F). The network analysis indicated that the overall degree of gut microbial connections was consistently increased in the ND group (Fig. 3G). Finally, we selected projects of NAFLD for cross-phenotype validation. Interestingly, the genera *Muribaculum* and uncultured *Bacteroidales bacterium* were consistently and significantly decreased in both obesity and NAFLD (Fig. 4A).

### 3.5 Case study II

Recent studies have shown that the gut microbial compositions and functions were significantly altered after bariatric surgery in severe obesity and this may be associated with its beneficial effects. Here, we collected three independent clinical studies on the microbial feature analysis following bariatric surgery that consisted of five inter-group comparisons according to the different time points in “Cross-project validation” module (Supplementary Table S2 and Supplementary Fig. S5). Following the analysis of “Cross-project validation” module, we identified that four genera *Veillonella*, *Akkermasia*, *Fusobacterium*, and *Haemophilus* were consistently and significantly increased after surgery within at least three comparisons (Supplementary Fig. S5A–C). Among which, *Veillonella, Akkermasia*, and *Fusobacterium* were also identified as a common microbial feature following surgery across multiple studies (Fouladi *et al.* 2021). Moreover, the *Firmicutes*/*Bacteroidetes* ratio was stable without significant changes while alpha diversity indices were recovered after surgery (Supplementary Fig. S5D and E). The function analysis further showed seven consistently downregulated metabolic pathways [e.g. Ko00785 (lipoic acid metabolism), Ko00600 (sphingolipid metabolism), and Ko00430 (taurine and hypotaurine metabolism)] and two upregulated metabolic pathways [i.e. Ko03010 (ribosome) and Ko0052 (galactose metabolism)] in five comparisons after surgery (Supplementary Fig. S5F). The network analysis of degree showed that *Bacteroides*, *Subdoligranulum*, *Faecalibacterium*, and *Balutia* were consistently prevalent in the networks after surgery (Supplementary Fig. S5F). More interestingly, we chose another group of metagenomics studies on drug intervention for cross-intervention validation, and further confirmed that *Akkermasia* and *Blautia* were consistently increased or decreased after different intervention strategies (Fig. 4B).

## 4 Discussion

OBMeta is developed with comprehensive capabilities including comparative metagenomics analysis, cross-project validation, cross-phenotype, and cross-intervention comparisons. To the best of our knowledge, this is the first versatile tool comprising metagenomics data processing, comparative analysis, co-abundance network comparison, and multidimensional validation, which aims to identify microbial features and biomarkers of obesity-associated metabolic diseases.

With increasing high-quality public metagenomics datasets from studies of metabolic diseases, researchers have started to integrate independent public datasets/projects with the same phenotype or intervention to explore the consistent microbial signatures for validation. For example, several 16S rRNA sequencing studies were integrated to evaluate the microbial changes of beneficial diets (peach, wheat, quinoa, barley, cherry, raspberry, and apple) in healthy and obese animal models (Garcia-Mazcorro *et al.* 2020). In addition, Fouladi *et al.* (2021) have characterized robust and consistent microbial signatures across multiple projects with obese patients after Roux-en-Y Gastric bypass (RYGB) surgery using their own and existing sequencing data. OBMeta can automatically conduct cross-project, cross-phenotype, and cross-intervention comparisons, facilitating the identification and validation of obesity-associated microbial features more accurately and efficiently.

Over the past decade, several web-based metagenomics comparative tools have been developed to contribute the exploration of gut microbial features and biomarkers of different diseases. EBI-Metagenomics (Hunter *et al.* 2014), VMPS (Huse *et al.* 2014), and MG-RAST (Wilke *et al.* 2016) are developed primarily for raw sequence processing and annotation, they also provide limited statistical methods and visualizations. Busybee (Schmartz *et al.* 2022) is a new server for metagenomic data analysis in the form of assembled contigs or long reads. For single project exploration of comparative metagenomics, the METAGEN-assist (Arndt *et al.* 2012) and Microbiome-Analyst (Lu *et al.* 2023) are representative tools comprising both univariate and multivariate statistical selections. Microbiome-Analyst 2.0 also integrates with more newly developed statistical algorithms, functional annotation and visualization, and even allows users to upload multiple comparisons for potential biomarker identification. Compared to Microbiome-Analyst, OBMeta evaluates the consistent or inconsistent alterations of gut microbial compositions and differential expressions in an efficient way by integrating public datasets of obesity-related metabolic diseases. More details of existing programs are summarized and compared in Supplementary Table S4.

The limitations of the current version of OBMeta deserve to be mentioned. First, in addition to obesity, T2D and NAFLD, other obesity-associated diseases like hypertension and hyperlipidemia which could also be associated with gut dysbiosis were not included due to the limited number of public accessible studies. So far, we have included 4283 samples spanning 90 projects, however, the curated database may not cover all the associated datasets available in the public datasets. In the future, our team will continually update more well-designed projects and obesity-associated diseases to enlarge the curated database. It is also highly recommended that users may contribute their own projects or notify us any publicly available projects of interest through webserver link. Besides, more new statistical algorithms will be integrated into OBMeta for biological interpretation. Ultimately, while the current OBMeta primarily addresses metabolic diseases related to obesity, the bioinformatic framework of integrating a specialized data analysis pipeline with a curated database for meta-analysis serves as a valuable model for other diseases benefiting from the growing availability of public resources in metagenomic studies, such as gastrointestinal cancers.

## 5 Conclusion

OBMeta is the first comprehensive web-based tool to analyze and validate the gut microbial signatures and biomarkers of obesity-associated metabolic diseases by streamlining the process of single-project analysis, cross-project validation, cross-phenotype, and cross-intervention comparisons.

## Supplementary Material

btad715_Supplementary_Data

## Data Availability

OBMeta is freely available at: http://obmeta.met-bioinformatics.cn/.
