# Supplementary material for: OBMeta: a comprehensive web server to analyze and validate gut microbial features and biomarkers for obesity-associated metabolic diseases
Source: Bioinformatics. 2023 Dec 11;39(12):btad715. doi: 10.1093/bioinformatics/btad715 (PMC10963062; doi:10.1093/bioinformatics/btad715)
Supplement: btad715_Supplementary_Data [file btad715_supplementary_data.docx]

Additional material

For manuscript titled “**OBMeta: a comprehensive web server to analyze and validate gut microbial features and biomarkers for obesity-associated metabolic diseases**” by Cuifang Xu, Jiating Huang, *et al*.

**1. Supplementary table**

**Supplementary Table 1.** A list of software or algorithms applied in data processing

| **Name** | **Version** | **Parameter** |
| --- | --- | --- |
| FastQC | 0.11.8 | Default |
| QIIME 2 | 2020.2 | Default |
| DADA 2 | 1.16.0 | Default |
| PICRUSt2 | 2.5.0 | Default |
| Trimmomatic | 0.39 | ILLUMINACLIP:TruSeq2-PE.fa:2:40:15  SLIDINGWINDOW:6:20 LEADING:3  TRAILING:3 MINLEN:50 |
| Bowtie 2 | 2.4.1 | Default |
| MEGAHIT | 1.2.9 | --min-contig-len 500 --min-count 2 |
| MetaGeneMark | 3.38 | -a -d -f G -m |
| CD-HIT | 4.8.1 | -c 0.95 -aS 0.9 -g 1 -d 0 -M 0 |
| Diamond | 0.9.34 | -e 1e-5 |
| Salmon | 1.2.1 | Default |

**Supplementary Table 2.** The projects used for cross-project validation and advanced validation

| **Filtering condition** | **Cross-project validation** | | **Advanced validation** | |
| --- | --- | --- | --- | --- |
| **Disease type** | Obesity | Obesity | NAFLD | Obesity |
| **Classification** | Phenotype | Intervention | Phenotype | Intervention |
| **Age stage** | Adult | - | Adult | Adult |
| **Gender** | Male | - | Male | Male |
| **Species type** | Mus musculus | - | Mus musculus | Mus musculus |
| **Sample type** | Feces | - | Feces | Feces |
| **Sequencing platform** | Illumina Miseq | - | Illumina Miseq | Illumina Miseq |
| **Intervention type** | - | Surgery | - | Drug |
| **Intervention effect** | - | Positive | - | Positive |
| **Projects** | PRJNA745524, PRJNA761909, PRJNA754322 | LAB001,  PRJNA395660,  PRJNA12042 | PRJEB36797, PRJEB48939, PRJDB7523 | PRJNA601832  PRJNA760837, PRJNA761909, PRJNA811511, PRJDB9484 |

**1.3 Supplementary Table 3 The accepted output datasets compatible with OBMeta**

|  | **16S** | **Metagenomics** |
| --- | --- | --- |
| **For compositional analysis** | QIIME2 (Bolyen, et al., 2019)/ Kraken2 (Wood, et al., 2019) | MetaPhlAn4 (Blanco-Miguez, et al., 2023)/ Kraken2 (Wood, et al., 2019) |
| **For functional analysis** | PICRUSt2 (Douglas, et al., 2020)/ Tax4Fun2 (Wemheuer, et al., 2020) | HUMAnN3 (Beghini, et al., 2021) |

**1.4 Supplementary Table 4 Comparison of OBMeta with other metagenomic comparative web servers**

| Web server | OBMeta | Microbiome-Analyst | METAGEN-assist | MG-RAST | Busybee | EBI-Metagenomics | VAMPs |
| --- | --- | --- | --- | --- | --- | --- | --- |
| Registration | Optional | No | No | Yes | No | No | Yes |
| Data type | 16S or metagenomic data | 16S or metagenomic data | 16S or metagenomic data | 16S or metagenomic data | Metagenomic data (binning contig) | 16S or metagenomic data | 16S or metagenomic data |
| Input | Count table/ BIOM | Count table/ BIOM/ mothur output | Count table/ BIOM/ mothur output/ QIIME output/ MG-RAST output/ MEGAN output/ STAMP output | Sequences | Sequences | Sequences | Sequences |
| Output of one project comparison |  |  |  |  |  |  |  |
| Alpha-diversity | Shannon and Simpson | Multiple | NA | Shannon | NA | NA | Multiple |
| Beta-diversity | PCoA and NMDS | PCoA and NMDS | PCA and PLS-DA | PCoA | NA | PCA | PCoA and NMDS |
| F/B ratio | Yes | NA | NA | NA | NA | NA | NA |
| Functional prediction | PICRUSt2 | PICRUSt and Tax4Fun | NA | NA | NA | NA | NA |
| Functional annotation | KEGG | COG and KEGG | NA | SEED, KEGG and COG | NA | GO | NA |
| Pathway visualization | Yes (SVG) | Yes (JavaScript) | NA | Yes (SVG) | NA | NA | NA |
| Co-abundance network construction | Yes | NA | NA | NA | NA | NA | NA |
| Network property calculation | Yes | NA | NA | NA | NA | NA | NA |
| Analysis application | Univariate methods/ LEfSe/ ANCOMBC/ DESeq2 | Univariate methods/ DESeq2/ edgeR/ metagenomeSeq/ Random Forests | Univariate methods/ SVM/ Random Forests | NA | Univariate methods | NA | NA |
| Output of cross-project validation |  |  |  |  |  |  |  |
| Alpha-diversity | Shannon and Simpson | Yes | NA | NA | NA | NA | NA |
| Beta-diversity | NA | Yes | NA | NA | NA | NA | NA |
| F/B ratio | Yes | NA | NA | NA | NA | NA | NA |
| Functional prediction | PICRUSt2 | NA | NA | NA | NA | NA | NA |
| Functional annotation | KEGG | NA | NA | NA | NA | NA | NA |
| Pathway visualization | Yes (SVG) | NA | NA | NA | NA | NA | NA |
| Network property calculation | Yes | NA | NA | NA | NA | NA | NA |
| Analysis application | Univariate methods/ LEfSe/ ANCOMBC/ DESeq2 | MaAsLin2 and MMUPHin | NA | NA | NA | NA | NA |
| Consistency evaluation with public project | Yes | NA | NA | NA | NA | NA | NA |
| Output of advance validation |  |  |  |  |  |  |  |
| Analysis application | Univariate methods/ LefSe/ ANCOMBC/ DESeq2 | NA | NA | NA | NA | NA | NA |
| Consistency evaluation with other related disease | Yes | NA | NA | NA | NA | NA | NA |
| Consistency evaluation with other interventions | Yes | NA | NA | NA | NA | NA | NA |

NA means none of this function.

The web servers are available at:

(1) OBMeta: <http://ob-meta.met-bioinformatics.cn/>

(2) MicrobiomeAnalyst: http://www.microbiomeanalyst.ca/

(3) METAGENassist: http://www.metagenassist.ca/

(4) MG-RAST: http://metagenomics.anl.gov/

(5) Busybee: <https://ccb-microbe.cs.uni-saarland.de/busybee-update>

(6) EBI-Metagenomics:https: //www.ebi.ac.uk/metagenomics/

(7) VAMPS: https://vamps2.mbl.edu/

**2. Supplementary Figure**

**Supplementary Figure 1**


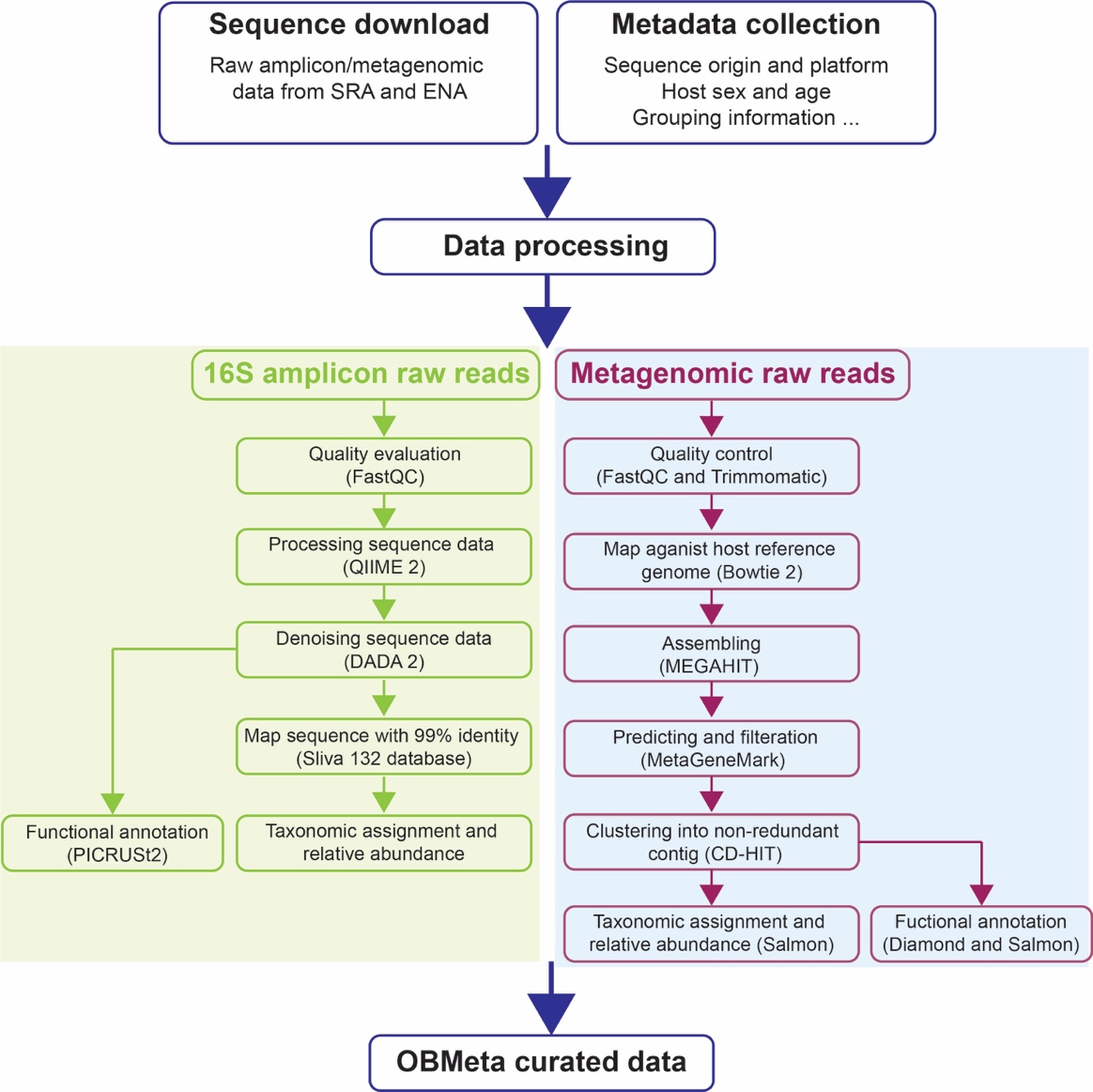


**Supplementary Figure 1.** The overall workflow of data processing for the curated database.

**Supplementary Figure 2**

**
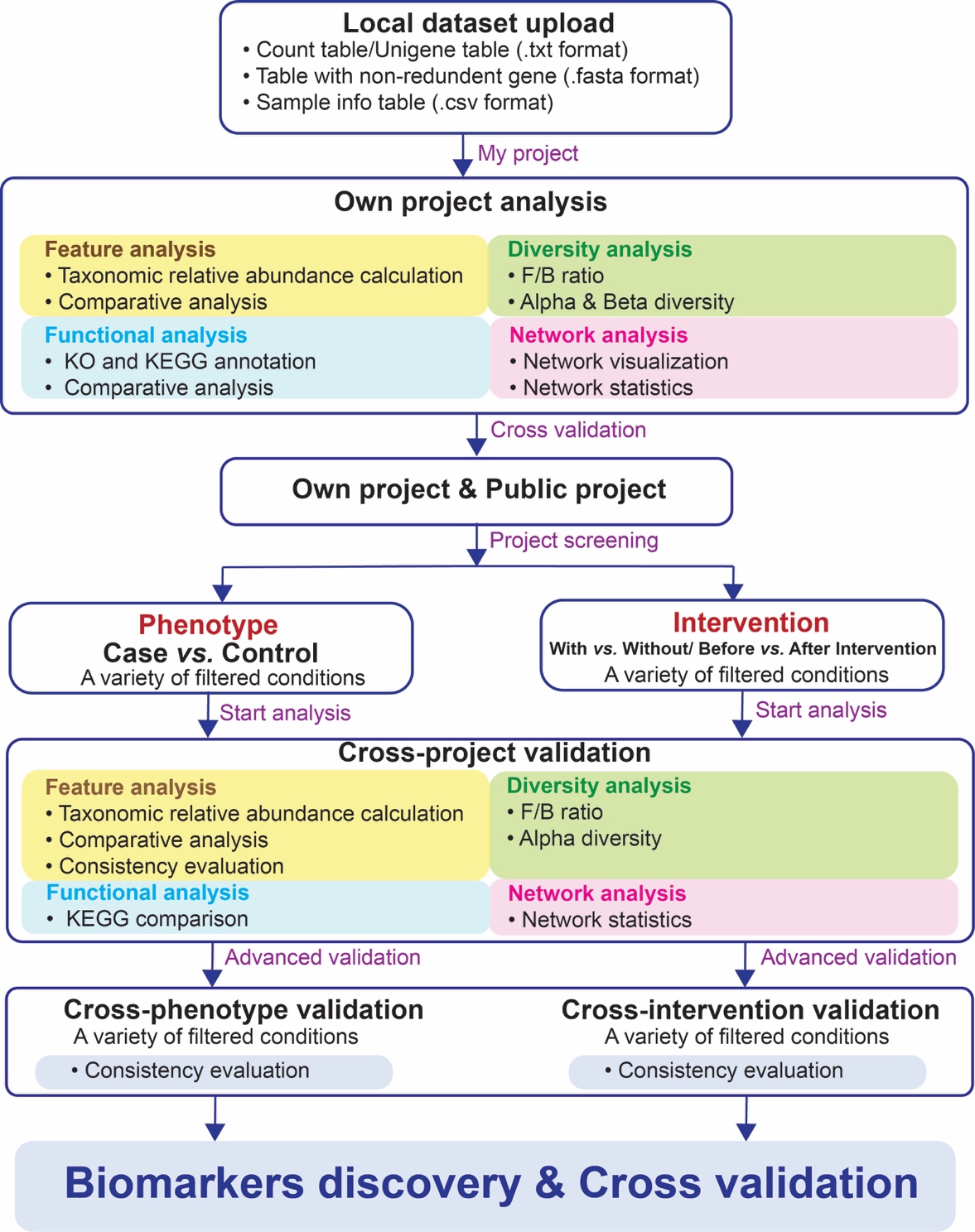
**

**Supplementary Figure 2.** The recommended analysis strategy using OBMeta.

**Supplementary Figure 3**

**
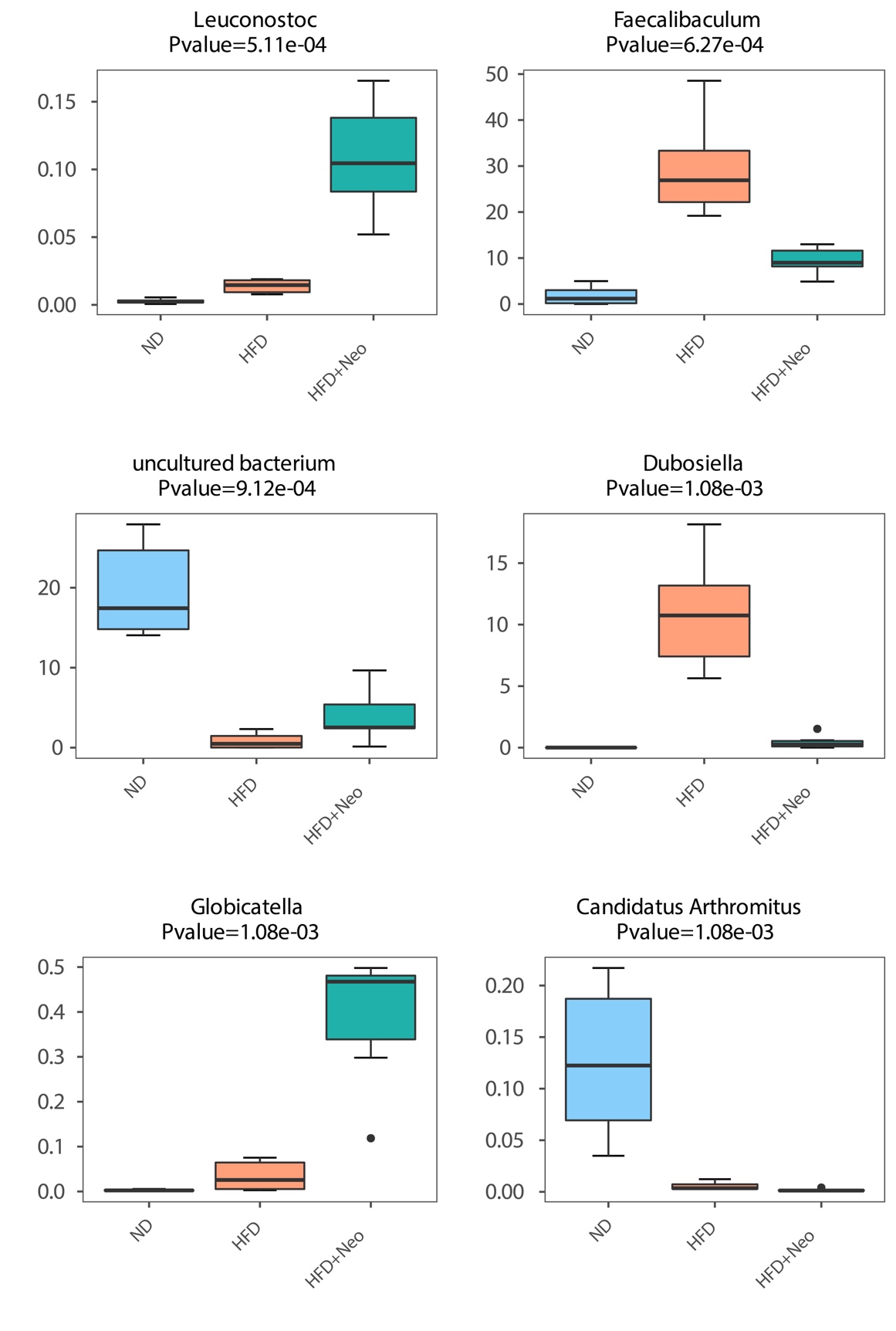
**

**Supplementary Figure 3.** One example output of the local dataset in feature analysis (at genus level). All genera will be compared in OBMeta and visualized in box plots in download file. In the real-time interface, OBMeta only presents top 6 most differentially abundant genera.

**Supplementary Figure 4**

**
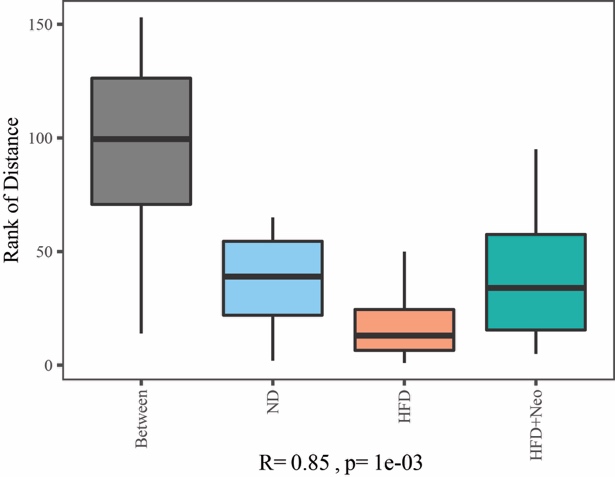
**

**Supplementary Figure 4.** Analysis of similarities (ANOSIM) test used in beta diversity comparison. The box plot shows the ranked dissimilarities between groups and the ranked dissimilarities within groups.

**Supplementary Figure 5**

**
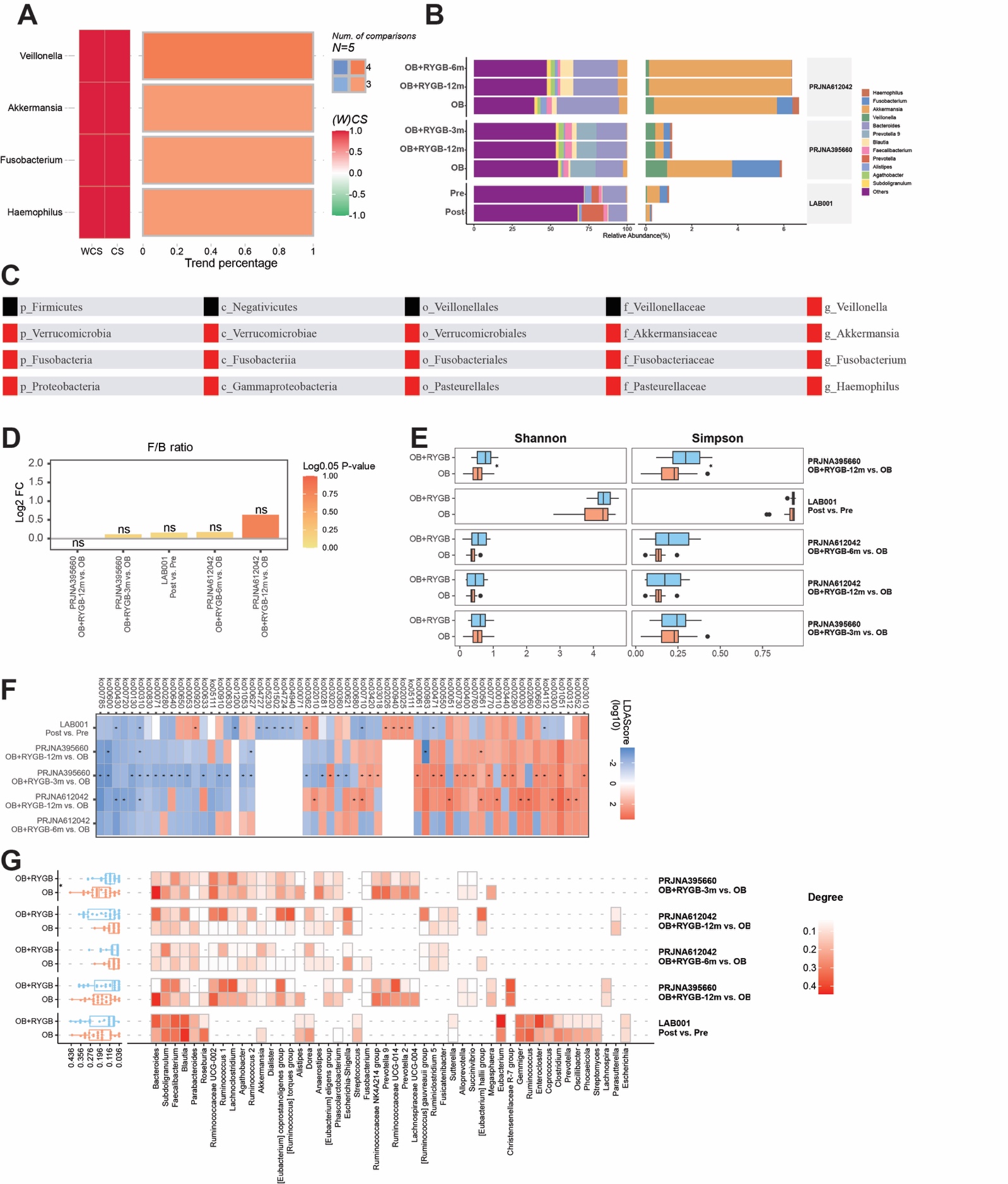
**

**Supplementary Figure 5.** Example outputs of the cross-project validation in ‘Intervention’ classification. **(A)** The consistency heatmap evaluated the intervention effect on gut microbiome at the genus level through multiple comparisons using default settings (comparison number = 3, consistency score = 0.6). **(B)** Sankey network of the consistently varied genus in selected projects. **(C)** The stacked bar plot of the relative abundance of all genera (the left panel) and the consistently varied genera (the right panel). **(D)** The log2 transformation of the fold change of the *Firmicutes*/*Bacteroidetes* ratio. ns: no significance. **(E)** Alpha diversity. It is measured by Shannon and Simpson indices using the genus profile. **(F)** The functional pathway with significant LDA score based on LEfSe analysis. **(G)** The comparison of the network property (degree) in each group. The network is constructed based on the top 30 most abundant genera. The heatmap shows the degree value of each genus in the network.

**References:**

Beghini, F.*, et al.* Integrating taxonomic, functional, and strain-level profiling of diverse microbial communities with bioBakery 3. *Elife* 2021;10.

Blanco-Miguez, A.*, et al.* Extending and improving metagenomic taxonomic profiling with uncharacterized species using MetaPhlAn 4. *Nat Biotechnol* 2023.

Bolyen, E.*, et al.* Reproducible, interactive, scalable and extensible microbiome data science using QIIME 2. *Nat Biotechnol* 2019;37(8):852-857.

Douglas, G.M.*, et al.* PICRUSt2 for prediction of metagenome functions. *Nat Biotechnol* 2020;38(6):685-688.

Wemheuer, F.*, et al.* Tax4Fun2: prediction of habitat-specific functional profiles and functional redundancy based on 16S rRNA gene sequences. *Environ Microbiome* 2020;15(1):11.

Wood, D.E., Lu, J. and Langmead, B. Improved metagenomic analysis with Kraken 2. *Genome Biol* 2019;20(1):257.
